# Development and Evaluation of Candied Pumpkin Produced with Jerusalem Artichoke Syrup

**DOI:** 10.3390/foods15142424

**Published:** 2026-07-08

**Authors:** Farida Smolnikova, Eleonora Okuskhanova, Kumarbek Amirkhanov, Bakhytkul Asenova, Almagul Nurgazezova, Gulnur Nurymkhan, Gulmira Mirasheva, Shujaul Mulk Khan, Sandugash Toleubekova, Diana Sviderskaya, Madina Jumazhanova

**Affiliations:** 1 Department of “Food Technologies”, Shakarim University, Semey 070000, Kazakhstan; f-smolnikova@mail.ru (F.S.); aspirant57@mail.ru (K.A.); b.asenova@shakarim.kz (B.A.); almanya1975@mail.ru (A.N.); gulnu-n@mail.ru (G.N.); gulmira_mir@mail.ru (G.M.); saltosha-sandu@mail.ru (S.T.); madina.omarova.89@mail.ru (M.J.); 2 Department of Ecology & Plant Sciences, Quaid-i-Azam University, Islamabad 15320, Pakistan; smkhan@gau.edu.pk; 3 Department of Biotechnology, Toraigyrov University, 64 Lomova Ave., Pavlodar 140000, Kazakhstan; sofilsev@rambler.ru

**Keywords:** osmotic dehydration, sucrose replacement, functional confectionery, water activity, texture, microbiological stability

## Abstract

This study developed candied pumpkin using Jerusalem artichoke syrup as an alternative to sucrose and evaluated its physicochemical properties, microstructure, sensory quality, and storage stability. Five formulations with Jerusalem artichoke syrup-to-pumpkin ratios of 1.25:1.00–1.30:1.00 were compared with a sucrose-based control. Syrup-based samples contained more protein (0.96–0.99 g/100 g) and fewer carbohydrates (21.25–23.35 g/100 g) than the control (0.40 and 40.40 g/100 g, respectively). Their caloric values ranged from 90.37 to 99.34 kcal/100 g, compared with 163.20 kcal/100 g in the control. Initial hardness was lower in syrup-based samples (30–40 kPa) than in the control (45 kPa), indicating a less rigid texture. Microstructural analysis showed preserved fibrous organization and a more uniform matrix, particularly in Sample 3 (1.27:1.00). This formulation also showed favorable sensory characteristics and higher contents of vitamin C, pantothenic acid, niacin, phosphorus, magnesium, sodium, and iron. During storage, moisture content decreased and water activity increased gradually but remained below 0.60. Microbial counts remained within permissible limits for 9 months, with no detectable coliforms or pathogenic microorganisms. Therefore, Jerusalem artichoke syrup may be considered a promising sucrose substitute for producing candied pumpkin with improved nutritional value and acceptable storage stability.

## 1. Introduction

Plant-based foods are fundamental in modern diets, providing carbohydrates, vitamins, and macro- and micronutrients that help maintain health [1,2]. Vegetables such as pumpkin (*Cucurbita maxima*) are especially valued for their robust nutritional profile, including vitamins A, C, E, K, and B vitamins, as well as important minerals and biologically active compounds such as carotenoids, phenolic compounds, flavonoids, and polysaccharides [2,3]. Owing to their low caloric content yet high fiber and antioxidant levels, pumpkins are frequently recommended as part of a balanced diet to support digestion, vision, and overall well-being [2,4].

Candied fruits, traditionally made by saturating fruits or vegetables with sugar, are common confectionery items used as fillings, dessert components, or decorations. This production process not only improves flavor but also extends shelf life [5]. Researchers have explored candied fruit made from pumpkins, carrots, and other vegetables using different sweetening and drying methods [6,7].

Pumpkin stands out among candied vegetable options because of its abundant bioactive substances, including carotenoids (provitamin A), which play an antioxidant role in the human body [8,9]. These compounds may help reduce oxidative stress, protect various organ systems, and even support immunomodulatory functions [4,10]. Pumpkin polysaccharides are particularly noteworthy: they have demonstrated antioxidant potential both in vitro and in vivo, highlighting their promise as ingredients for functional foods [11,12,13]. In addition, certain pumpkin extracts appear to slow cancer cell growth [14] and may offer hypoglycemic properties useful in managing diabetes [15].

Despite pumpkins’ nutritional advantages, conventional candied products often rely on substantial amounts of beet or cane sugar, raising concerns for consumers who need to limit sugar intake, including individuals at risk of diabetes or obesity [16]. The World Health Organization recommends reducing free sugar intake throughout the life course and limiting free sugars to less than 10% of total energy intake, with a further reduction to below 5% suggested for additional health benefits [17]. In diabetes care, the American Diabetes Association emphasizes individualized nutrition therapy and evidence-based dietary patterns to improve glycemic control and support body-weight management [18]. Therefore, reducing sucrose content in candied fruits is consistent with current public-health and diabetes-nutrition recommendations.

Jerusalem artichoke (*Helianthus tuberosus* L.) tubers are characterized by high moisture content and a dry-matter fraction rich in carbohydrates, especially fructan-type polysaccharides such as inulin. They also contain dietary fiber, protein, ash, low levels of fat, and minerals such as calcium and phosphorus [19,20]. This composition explains the interest in Jerusalem artichoke as a raw material for low-fat, fiber-containing, and prebiotic food ingredients. Despite the potential of Jerusalem artichoke syrup as a functional sweetening agent, its application in candied pumpkin production has not been sufficiently studied. To address these needs, this study proposes replacing sugar syrup with Jerusalem artichoke syrup in the production of candied pumpkin. Jerusalem artichoke syrup has a low GI of about 13–16, meaning it is metabolized more gradually and produces smaller fluctuations in blood glucose than standard sugar [21]. By using this syrup, the resultant candied pumpkin may be suitable for individuals who want or need to reduce their refined sugar intake, including those concerned with weight management and glycemic control [16].

The species *Cucurbita maxima* Duch was selected as the raw material due to its wide availability, low cost, and well-documented nutritional value. This type of pumpkin is extensively cultivated in Kazakhstan, and its flesh provides a balanced composition of water, carbohydrates, protein, and dietary fiber [22]. Therefore, pumpkin represents a suitable nutrient-rich raw material for confectionery applications.

In candied fruit technology, the choice of syrup is important because the process is based on osmotic dehydration, in which a hypertonic solution promotes water removal and solute uptake. Sucrose syrup remains the most widely used osmotic medium; however, glucose, fructose, invert sugar, corn syrup, honey, and concentrated fruit juices have also been reported as osmotic agents for fruit and vegetable processing [23,24]. These sweetening systems can influence dehydration kinetics, sweetness, color, flavor, texture, and final water activity. Nevertheless, most of them mainly provide readily digestible sugars and do not contribute a significant prebiotic component. Jerusalem artichoke syrup differs from these conventional syrups because it is derived from *Helianthus tuberosus* tubers, which are rich in inulin and fructooligosaccharides. Thus, its use may combine technological functionality as an osmotic agent with potential nutritional advantages compared with conventional sugar-based syrups.

The originality of the present study lies in the application of Jerusalem artichoke syrup as a sucrose substitute in candied pumpkin and in the comprehensive evaluation of the resulting product, including chemical composition, caloric value, microstructure, hardness, moisture retention, water activity, sensory quality, and microbiological stability during storage. The goal of this work was to study the effect of Jerusalem artichoke syrup on the nutritional, physicochemical, sensory, structural, and safety characteristics of candied pumpkin products.

## 2. Materials and Methods

### 2.1. Sample

Pumpkin (*Cucurbita maxima Duch*) was delivered from the farm “Pryrechnoye” (Semey, Kazakhstan). Jerusalem artichoke syrup was obtained from the company brand “Bionational”—“Natural Jerusalem artichoke syrup”, (G El Bio LLC, Moscow, Russia). Sugar was delivered from the Aksu sugar factory, (Zhansugurov settlement, Aksu district, Almaty region).

### 2.2. Pumpkin Candied Fruits with Jerusalem Artichoke Syrup Production

Candied pumpkin was produced from the Winter Sweet variety of *Cucurbita maxima* Duch. Fresh pumpkins were washed, peeled, cut, and blanched before further processing. Losses during washing and peeling were approximately 22%, while losses during blanching were approximately 17%. The quantitative formulations of the samples are presented in Table 1.

Five experimental formulations were prepared by varying the mass ratio of Jerusalem artichoke syrup to peeled pumpkin from 1.25:1.00 to 1.30:1.00 (*w*/*w*). This range was selected based on preliminary technological trials and practical requirements for candied fruit production. Ratios below 1.25:1.00 did not provide sufficient syrup for uniform impregnation of pumpkin pieces, whereas higher syrup levels increased surface stickiness, deformation, and the intensity of Jerusalem artichoke syrup flavor. Therefore, the selected range was considered suitable for evaluating the effect of small changes in syrup-to-pumpkin ratio on the quality of candied pumpkin.

Natural Jerusalem artichoke syrup was used without dilution. According to the manufacturer’s specification, the syrup contained 66 g of carbohydrates and 0.01 g fat per 100 g, with an energy value of 271 kcal/100 g. Therefore, the carbohydrate concentration of the Jerusalem artichoke syrup was considered to be 66% (*w*/*w*).

A sucrose-based control was prepared from the same batch of pumpkin. The control formulation contained 1000 g peeled pumpkin, 720 g sucrose, and 600 g water. The sucrose concentration in the control syrup was calculated using the following Equation (1):

*Sucrose concentration* (%) = [*mass of sucrose*/(*mass of sucrose* + *mass of water*)] × 100 (1)

Accordingly, the sucrose concentration in the control syrup was 54.5% (*w*/*w*). The control and experimental samples were produced under identical processing conditions, including cutting, blanching, soaking, drying, packaging, and storage. The only difference between the formulations was the osmotic agent: Jerusalem artichoke syrup was used in the experimental samples, while sucrose syrup was used in the control.

#### Technology for Producing Candied Pumpkin with Jerusalem Artichoke Syrup

**Figure 1:**
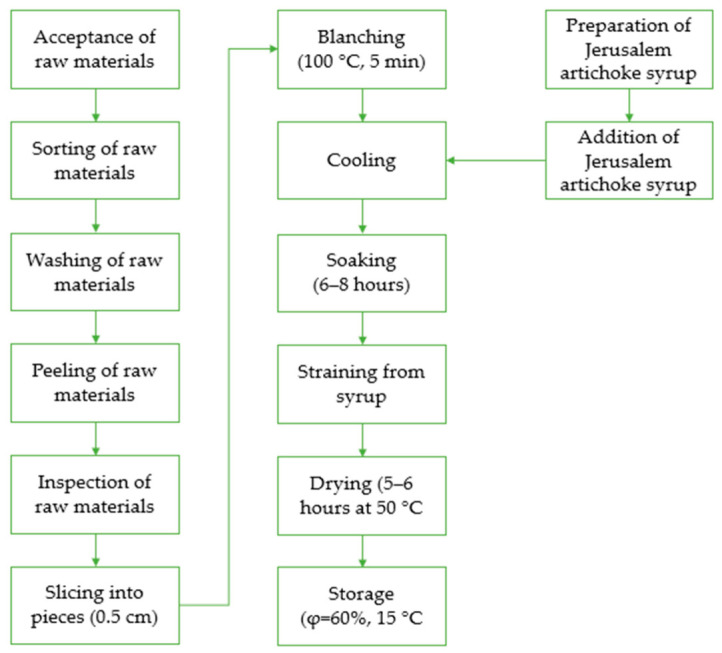
Technological scheme of production of candied pumpkin with Jerusalem artichoke syrup.

Figure 1 shows the process flow for producing candied pumpkin using Jerusalem artichoke syrup. First, fresh pumpkin (*Cucurbita maxima Duch*) was washed and peeled; the flesh was cut into cubes (0.5–1 cm) with a Bosch MUM 58252 (Robert Bosch GmbH, Stuttgart, Germany) food processor. The pumpkin cubes were blanched in boiling water for 5 min in a standard 5 L saucepan on a Bosch HKA-050050Q (Robert Bosch GmbH, Stuttgart, Germany) glass-ceramic electric stove. Blanching deactivates enzymes, prevents browning, and increases cell permeability, which helps the pumpkin absorb syrup more effectively. After blanching, the cubes were cooled in cold water and spread on a baking sheet to remove excess moisture.

Jerusalem artichoke syrup was added at ratios ranging from 1.25:1.00 to 1.30:1.00 (syrup:pumpkin, *w*/*w*). The pumpkin cubes were left to soak in the syrup for 6–8 h at 18–20 °C to ensure thorough impregnation. After soaking, the excess syrup was drained using lattice molds (cell diameter 5–7 mm), allowing the pumpkin pieces to drip completely. The drained syrup was reserved for making subsequent batches or other sweet products.

Next, the syrup-infused pumpkin cubes were placed in a single layer on perforated baking sheets and loaded into the Tribest Sedona Combo SD-P9150 (Tribest Corporation, Anaheim, CA, USA) dehydrator. A gentle drying mode was applied (55 °C) for about 5–6 h, until the final moisture content reached approximately 20.1–23.1%. Drying conditions were controlled to minimize color degradation and nutrient losses in the pumpkin. The finished candied pumpkin was cooled to 18–20 °C and stored at ≤15 °C with a relative humidity of about 60%.

### 2.3. Determination of Selected Raw-Material Characteristics

Total phenolic content was determined using the Folin–Ciocalteu method described in [25]. Briefly, homogenized raw-material samples were extracted with 80% methanol at a sample-to-solvent ratio of 1:10 (*w*/*v*). The extract was filtered, and the filtrate was used for analysis. The extract was mixed with Folin–Ciocalteu reagent and sodium carbonate solution, incubated at room temperature for 2 h, and absorbance was measured at 760 nm. Gallic acid was used for calibration, and results were expressed as mg gallic acid equivalents (GAE)/100 g.

Dietary fiber content was determined by the enzymatic–gravimetric method. Samples were sequentially treated with α-amylase, protease, and amyloglucosidase, and the fiber fraction was precipitated with ethanol, dried, and weighed [26]. The analysis was performed using a VELP FIWE-3 fiber analyzer (VELP, Usmate, Italy), and results were expressed as g/100 g.

Inulin content in Jerusalem artichoke raw material was determined spectrophotometrically according to [27]. Inulin was extracted from the sample, hydrolyzed under acidic conditions, and quantified based on absorbance measurement.

The density of fresh pumpkin flesh was determined by the water displacement method, while the density of Jerusalem artichoke syrup and sucrose syrup was measured using a calibrated pycnometer at 20 ± 0.5 °C [28].

Viscosity was measured with a BOYN digital viscometer (BOYN Instrument Co., Ltd., Shanghai, China). Each sample was equilibrated at 18–22 °C, and approximately 100 mL was analyzed at 6 rpm for 60 s. Viscosity and shear stress were recorded directly from the instrument display. Measurements were performed in triplicate.

### 2.4. Determination of Water Activity

Water activity (a_w_) was measured using an aWLife analyzer (Steroglass S.r.l., Perugia, Italy). Before each series of measurements, the instrument was calibrated using standard reference solutions. Representative portions of candied pumpkin were placed in the measuring chamber and equilibrated at 25 °C. The a_w_ value was recorded automatically after equilibrium conditions had been reached. Measurements were performed in triplicate for each formulation on day 0 and after 1, 3, 6, and 9 months of storage. Samples stored in hermetically sealed packaging at 10–12 °C, 20–22 °C, and 30–32 °C were analyzed separately. The instrument was regularly calibrated to ensure the reliability and reproducibility of the results [29].

### 2.5. Determination of Proximate Composition

The proximate composition of candied pumpkin samples was determined using standard analytical procedures. Moisture content was determined according to ISO 1026:1982 [30]. Protein content was determined by the Kjeldahl method according to ISO 1871:2009 [31], and the nitrogen content was converted to protein using the appropriate conversion factor. Fat content was determined gravimetrically after hydrolysis and solvent extraction according to the principles of ISO 11085:2015 [32]. Total ash was determined gravimetrically by incineration to constant mass. Carbohydrate content was calculated by difference using the following Equation (2):

*Carbohydrates* (%) = 100 − [*moisture* (%) + *protein* (%) + *fat* (%) + *ash* (%)] (2)

### 2.6. Hardness Determination

The hardness of candied pumpkin samples was determined using an S 165 KIT cone dial penetrometer (PEL LLC, St. Petersburg, Russia). Measurements were carried out at a sample temperature of 20 ± 2 °C. Candied fruits of identical size and shape were selected to ensure uniform testing conditions.

A standard 30° cone with a 50 g load was used. Each sample was placed on the flat base of the penetrometer, after which the cone was gently lowered onto the surface of the candied fruit. The device was activated, and penetration was allowed for 5–10 s. The penetration depth (h, mm) was recorded for each measurement. At least five measurements were performed on different pieces from each sample batch, and the mean value was calculated.

The applied force (F, N) was calculated according to Equation (3):

*F* = *m*·*g* (3)

where *m* is the mass of the load (kg), *g* is gravitational acceleration (9.81 m/s^2^).

Hardness (σ, kPa) was determined as compressive stress using (Equation (4)):

*σ* = *F*/*A* (4)

where *A* is the contact area (m^2^) corresponding to the lateral surface area of the cone up to penetration depth *h*.

The contact area was calculated geometrically for a 30° cone based on the measured penetration depth. Hardness was evaluated on the first day after production and during storage (1, 3, 6, and 9 months). Samples were sealed, packaged and stored at 20 °C and relative humidity 60% until analysis.

### 2.7. Determination of Organoleptic Parameters of Candied Fruits

The organoleptic evaluation of candied pumpkin samples was conducted according to the general principles of sensory profiling and quantitative sensory assessment described in ISO 13299 [33] and the national standard for organoleptic evaluation of fruit and vegetable products [34]. The evaluated attributes included appearance, color, smell, consistency, taste, and overall acceptability. The evaluation panel consisted of 20 assessors, including 15 employees of the Food Safety laboratory and 5 external consumers. Before participation, assessors confirmed that they had no olfactory disorders, color-vision impairment, food allergies, or dietary restrictions related to pumpkin, Jerusalem artichoke syrup, or sucrose-containing products. All participants provided informed consent. The sensory evaluation was performed in the Food Safety laboratory under controlled conditions. The room was clean, well-ventilated, free from foreign odors, and provided sufficient lighting for visual assessment. Assessors were seated separately and evaluated the samples individually. Communication between assessors was not allowed during the tasting session. Candied pumpkin samples were served at 18–20 °C in portions of 15 g. Each sample was coded with a random three-digit number and presented in randomized order to minimize order bias. Drinking water was provided for palate cleansing between samples, and a 1–2 min interval was maintained between evaluations. The total evaluation time did not exceed 30–40 min.

Each attribute was assessed using a structured five-point scale, where 5 indicated the highest quality and 1 indicated the lowest quality. Assessors recorded their scores on individual evaluation sheets. The results were expressed as mean scores for each attribute and used to compare the sensory quality of the experimental formulations and the sucrose-based control.

### 2.8. Microstructural Analysis

The microstructure of the candied pumpkin samples was examined using a JSM-6390LV scanning electron microscope (JEOL Ltd., Tokyo, Japan) equipped with an INCA ENERGY 250 X-ray microanalysis system (Oxford Instruments, Abingdon, UK). Before analysis, representative fragments of each candied pumpkin sample were selected and prepared for microscopic examination. Images were obtained at an accelerating voltage of 10 kV and a magnification of ×500. The micrographs were evaluated to identify differences in tissue integrity, fiber organization, intercellular spaces, surface porosity, air inclusions, and the distribution of sugar crystals. The sugar-based control and the five samples produced with Jerusalem artichoke syrup were analyzed under identical conditions to ensure valid comparison of their microstructural characteristics.

### 2.9. Determination of Vitamins

Vitamin C, β-carotene, thiamine (B1), riboflavin (B2), pyridoxine (B6), and niacin were determined by HPLC using a Shimadzu LC-20 Prominence system (Shimadzu, Kyoto, Japan) equipped with UV–visible and fluorescence detectors. Separation was performed on a SUPELCO C18 column (250 mm × 4.6 mm). Certified vitamin standards were purchased from Sigma-Aldrich (St. Louis, MO, USA). Results were expressed as mg/100 g of product, and all analyses were performed in triplicate.

Vitamin C was determined according to the principles of EN 14130:2003 [35]. Homogenized samples were extracted with metaphosphoric acid solution, treated with L-cysteine to reduce dehydroascorbic acid to ascorbic acid, filtered through a 0.45 µm membrane filter, and analyzed by HPLC with detection at 265 nm. β-Carotene was determined separately according to EN 12823-2:2000 [36]. Samples were saponified with alcoholic potassium hydroxide in the presence of an antioxidant, extracted with an organic solvent under reduced light exposure, evaporated at ≤50 °C, redissolved in an HPLC-compatible solvent, and detected at 450 nm.

Thiamine, riboflavin, pyridoxine, and niacin were determined by reversed-phase HPLC according to the relevant EN methods [36,37,38,39,40]. Homogenized samples were extracted or hydrolyzed according to the target vitamin, centrifuged for 4–5 min at 7000–8000 rpm, diluted to 25 mL, and filtered through a 0.45 µm membrane filter. Mobile phase A was 0.6% phosphoric acid solution, pH 1.5–1.8, and mobile phase B was HPLC-grade acetonitrile. Gradient elution was performed at 0.8 mL/min with a 20 µL injection volume and a column temperature of 20 °C. Quantification was carried out using external calibration curves.

### 2.10. Determination of Mineral Composition

The mineral composition of candied pumpkin samples was determined by inductively coupled plasma mass spectrometry (ICP-MS). Homogenized samples (1–2 g) were placed in high-pressure Teflon vessels, ashed in a muffle furnace at 400 °C for 4 h and then at 600 °C for 2 h. One gram of ash was digested with 3 mL HNO_3_ and 2 mL HF using a Berghof SpeedWave microwave digestion system (Berghof Products + Instruments GmbH, Eningen, Germany) at 200 °C for 20 min. The digests were diluted to 10 mL with 1% HNO_3_.

Potassium, calcium, magnesium, sodium, sulfur, phosphorus, chlorine, and iron were quantified using a Varian 820-MS ICP-MS system (Varian Inc., Melbourne, VIC, Australia). The instrument was calibrated with certified multielement standards (Var-TS-MS and IV-ICPMS-71A; Inorganic Ventures, Christiansburg, VA, USA). Accuracy was verified using certified reference materials; deviations from certified values were below 10%. All measurements were performed in triplicate, and results were expressed as mg/100 g of product.

### 2.11. Microbiological Analysis

Microbiological analysis of candied pumpkin samples was carried out to determine total aerobic mesophilic count (TAMC), coliform bacteria, yeasts and molds, and pathogenic microorganisms, including *Salmonella* spp. The analyses were performed according to the relevant standard methods.

TAMC was determined according to State Standard 33536-2015 [41]. Appropriate serial dilutions of the samples were inoculated into agar nutrient medium and incubated aerobically at 30 ± 1 °C for 72 ± 3 h. Colonies were counted, and the results were expressed as CFU/g. Coliform bacteria were determined according to State Standard 31747-2012 [42] by inoculating the sample or its dilutions into selective lactose-containing liquid media, followed by incubation and confirmation of positive tubes based on gas formation and subsequent reseeding. Yeasts and molds were determined according to State Standard 10444.12-2013 [43]. The inoculated plates were incubated aerobically at 25 ± 1 °C for 5 days. Preliminary colony counting was performed after 3 days, and final counting was performed after 5 days. Yeast and mold colonies were differentiated visually and, when necessary, confirmed microscopically. Results were expressed as CFU/g.

Pathogenic microorganisms, including Salmonella spp., were determined according to Methodological Guidelines 4.2.2723-10 [44]. Prepared sample dilutions were inoculated onto selective agar media, including bismuth-sulfite agar and other differential diagnostic media, and incubated at 37 ± 1 °C for 18–24 h; bismuth-sulfite agar plates without visible growth were incubated for up to 48 ± 2 h. Suspicious colonies were subjected to further biochemical identification. Results were reported as detected/not detected in the specified sample mass.

### 2.12. Statistical Analysis

All experiments were independently repeated three times using three separate production batches. For each batch, analytical measurements were performed in technical replicates, and the technical replicate values were averaged before statistical analysis. The independent production batch was considered the experimental unit (*n* = 3), and results are presented as mean ± standard deviation.

The ANOVA model was selected according to the experimental design of each dataset. For variables measured at a single time point, formulation was the only fixed factor; therefore, differences among samples were analyzed using one-way ANOVA. For hardness and moisture content measured during storage, formulation and storage time were used as fixed factors, and the data were analyzed using two-way ANOVA. For water activity, formulation, storage temperature, and storage time were included as fixed factors because this parameter was evaluated under different temperature conditions; therefore, three-way ANOVA was applied. When significant effects were detected, Tukey’s honestly significant difference (HSD) test was used for multiple comparisons. Differences were considered statistically significant at *p* < 0.05. Statistical analyses were performed using XLSTAT 2020 software (Addinsoft Inc., Paris, France).

## 3. Results and Discussion

### 3.1. Characterization of Raw Materials

The chemical and physicochemical characteristics of the raw materials are presented in Table 2 and Table 3. Fresh pumpkin contained 87.1 g/100 g moisture, 12.3 g/100 g dry matter, 8.63 g/100 g carbohydrates, 1.33 g/100 g protein, 0.78 g/100 g ash, and 1.20 g/100 g dietary fiber. Its low energy value, 30 kcal/100 g, confirms its suitability as a low-calorie vegetable raw material for candied fruit production.

Jerusalem artichoke syrup differed markedly from fresh pumpkin and sucrose syrup. It contained 73.0 g/100 g dry matter, 65.8 g/100 g carbohydrates, 5.0 g/100 g inulin/fructans, and 2.0 g/100 g ash. Compared with sucrose syrup, Jerusalem artichoke syrup had slightly lower carbohydrate content and energy value, but higher ash content, acidity, soluble solids, and viscosity. Its viscosity was 3982 mPa·s, more than twice that of sucrose syrup. These properties may explain the stronger osmotic effect, improved moisture retention, and softer structure observed in the candied pumpkin samples produced with Jerusalem artichoke syrup.

The bioactive and mineral profile of the raw materials also helps explain the composition of the finished product. Fresh pumpkin was the main source of β-carotene, total phenolic compounds, potassium, calcium, magnesium, and iron [25]. In contrast, Jerusalem artichoke syrup contained a high level of vitamin C and phosphorus, reaching 93.8 mg/100 g and 79.9 mg/100 g, respectively. Therefore, the use of Jerusalem artichoke syrup could contribute to the higher vitamin C and phosphorus contents. Overall, the raw-material analysis confirms that the quality changes in the finished candied products were associated not only with dehydration and osmotic impregnation but also with the distinct composition of Jerusalem artichoke syrup.

### 3.2. Chemical Composition of the Candied Pumpkins

The replacement of sucrose syrup with Jerusalem artichoke syrup markedly changed the chemical profile of candied pumpkin (Table 4). The main effect was a substantial reduction in carbohydrate content and energy value. Compared with the sucrose-based control, the syrup-based formulations contained approximately 42–47% less carbohydrates and 39–45% fewer calories (*p* < 0.001). This difference can be explained not only by the lower carbohydrate concentration of Jerusalem artichoke syrup compared with sucrose syrup, but also by its different carbohydrate profile. Jerusalem artichoke syrup contains fructan-type carbohydrates, including inulin, which distinguishes it from sucrose syrup and may affect osmotic mass transfer, sugar uptake, and water retention in pumpkin tissue [45,46,47].

The slightly higher protein content in the Jerusalem artichoke syrup formulations also reflects the different composition of the osmotic medium and raw materials. Although the absolute protein level remained low, all experimental samples contained significantly more protein than the control (*p* < 0.001). Jerusalem artichoke tubers contain protein and amino acids, including arginine, glycine, aspartic acid, and glutamic acid [45]. Fat content was minimal in all syrup-based samples and was not nutritionally meaningful, although statistical differences were detected among formulations (*p* < 0.001). Ash content did not differ significantly among samples (*p* > 0.25), indicating that replacement of sucrose syrup did not substantially alter the total mineral residue of the finished product.

Moisture content was also affected by formulation (*p* < 0.01). Samples prepared with higher syrup-to-pumpkin ratios retained more moisture, which may be related to the high soluble-solids content, viscosity, and water-binding capacity of Jerusalem artichoke syrup components. Inulin and oligofructose are widely used as functional food ingredients because they can modify texture, improve mouthfeel, and partially replace sugar or fat in reduced-calorie formulations [46,47]. These properties may partly explain the softer texture and moisture-retention behavior observed in the syrup-based samples.

Overall, Jerusalem artichoke syrup acted not only as a sweetening and osmotic agent but also as a formulation component that changed the balance between sugar uptake, water retention, and energy value. The most important compositional outcome was the production of candied pumpkin with substantially lower carbohydrate and caloric content while maintaining comparable moisture and ash levels.

### 3.3. Microstructural Characteristics of Candied Pumpkin Produced with Jerusalem Artichoke Syrup

**Figure 2:**
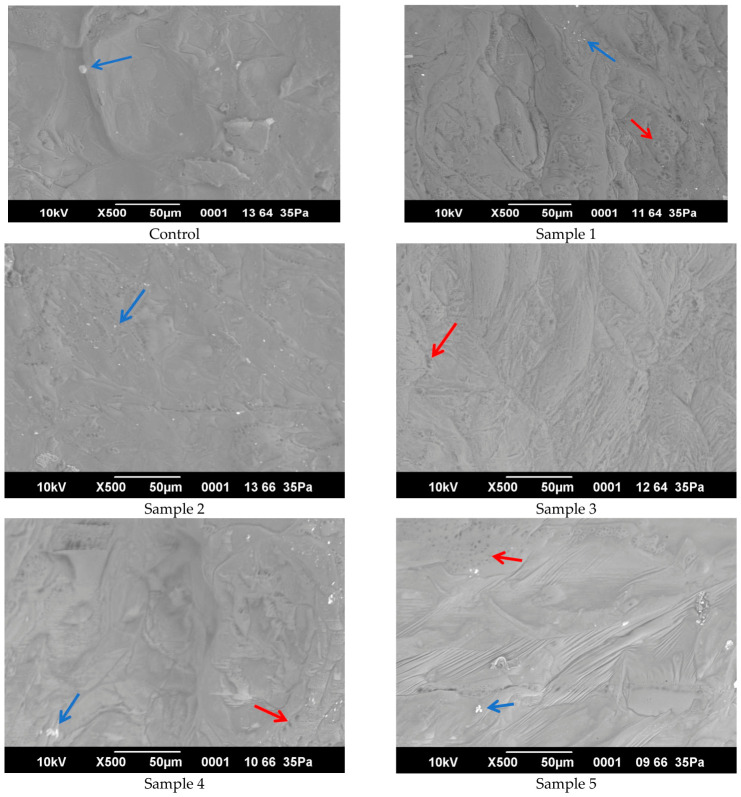
Microstructure of experimental and control samples of candied fruit (crystalline deposits are indicated by blue arrows, and pores or voids are indicated by red arrows).

Microstructural analysis revealed differences between candied pumpkin samples produced with Jerusalem artichoke syrup and the sucrose-based control (Figure 2). The control sample exhibited a dense and compact tissue matrix with closely connected cellular structures. Cell-wall integrity was largely preserved, while discrete crystalline deposits were visible within the matrix and on the surface. The compact structure may be associated with osmotic impregnation and subsequent drying, which promote solute accumulation and strengthen intercellular adhesion. Similar structural changes have been reported during diffusion and dehydration processes in pumpkin tissues [48].

Samples produced with Jerusalem artichoke syrup differed in porosity, distribution of crystalline deposits, and organization of the fibrous matrix. Sample 1 showed tightly packed tissues with visible pores or voids distributed throughout the structure and several crystalline deposits. Sample 2 also presented a dense matrix, but smaller crystalline deposits appeared more frequently, while the number of visible voids was comparatively lower.

Sample 3 exhibited the most homogeneous microstructure among the experimental formulations. Small pores or voids were evenly distributed within the matrix, and no prominent crystalline deposits were observed in the selected field of view. The fibrous structure appeared continuous and well-integrated, indicating a more uniform organization of the tissue. This structural pattern may reflect a balanced rate of solute diffusion and moisture removal during osmotic impregnation and drying, as previously described for fruit matrices subjected to osmotic treatment [49].

Samples 4 and 5 displayed dense fibrous structures with a limited number of visible pores or voids. Sample 4 exhibited a relatively coarse and compact matrix with occasional crystalline deposits. Sample 5 showed elongated fibers and several larger crystalline deposits. The observed differences suggest that the syrup-to-pumpkin ratio influenced tissue porosity, solute distribution, and matrix densification. Higher osmotic-solute concentrations can increase solid gain and promote a denser structure during dehydration [50]. Interactions between low-molecular-weight carbohydrates and pumpkin polysaccharides may also enhance intermolecular cohesion through hydrogen bonding [51].

All experimental samples retained a recognizable fibrous structure and substantial intercellular adhesion, indicating that impregnation with Jerusalem artichoke syrup followed by gentle drying preserved the general architecture of pumpkin tissue. Osmotic dehydration before drying can reduce structural collapse and support the retention of product shape and texture [52]. Compared with the more compact control matrix, the samples produced with Jerusalem artichoke syrup generally exhibited a more plasticized structure, with Sample 3 showing the most uniform organization.

Overall, Jerusalem artichoke syrup affected the microstructure of candied pumpkin by modifying porosity, matrix compactness, and the distribution of crystalline deposits. Sample 3, prepared at a syrup-to-pumpkin ratio of 1.27:1.00, demonstrated the most homogeneous and stable microstructure, which is consistent with its favorable textural and organoleptic properties.

### 3.4. Hardness of Candied Pumpkin Produced with Jerusalem Artichoke Syrup

The hardness of freshly prepared candied pumpkin samples ranged from 30 to 45 kPa (Table 5). The sucrose-based control showed the highest initial hardness (45 kPa) and differed significantly from all formulations produced with Jerusalem artichoke syrup (*p* < 0.05). Among the experimental samples, Samples 1 and 2 showed the highest initial hardness values, at 40 and 38 kPa, respectively, with no significant difference between them (*p* ≥ 0.05). Samples 3 and 4 had intermediate hardness values of 35 and 34 kPa and also did not differ significantly from each other (*p* ≥ 0.05). Sample 5 showed the lowest initial hardness (30 kPa) and differed significantly from the other formulations (*p* < 0.05). These results indicate that decreasing the syrup-to-pumpkin ratio was associated with a gradual reduction in structural rigidity.

The lower hardness of the samples produced with Jerusalem artichoke syrup may be related to differences in carbohydrate composition and crystallization behavior compared with the sucrose-based control. Sucrose crystallization can promote the formation of rigid structural bridges within the food matrix, whereas reducing sugars generally crystallize less readily. Cell-wall integrity and pectin-mediated intercellular adhesion also contribute to the firmness of plant tissues [53,54].

Hardness increased during storage in all formulations. The control remained the hardest sample throughout the 9 months, increasing from 45 to 55 kPa (*p* < 0.05). Sample 1 increased from 40 to 47 kPa, while Sample 2 increased from 38 to 45 kPa. Samples 3 and 4 increased from 35 to 44 kPa and from 34 to 42 kPa, respectively. Sample 5 remained the softest formulation throughout storage, although its hardness increased from 30 to 40 kPa (*p* < 0.05).

The differences among formulations remained evident during storage. At months 0, 1, and 3, Samples 1 and 2 formed the group with the highest hardness among the experimental formulations, Samples 3 and 4 showed intermediate values, and Sample 5 had the lowest values (*p* < 0.05). At month 6, Sample 4 did not differ significantly from Samples 3 or 5, while at month 9, overlapping significance groups indicated a more gradual distribution of hardness values among the experimental formulations. Nevertheless, the control remained significantly harder than all syrup-based samples at each storage time (*p* < 0.05).

The progressive increase in hardness is consistent with moisture migration and sugar crystallization during storage, which can promote structural compaction in intermediate-moisture foods [55,56]. Osmotic dehydration agents influence firmness through solute gain, moisture reduction, and changes in matrix organization [57,58]. Overall, Sample 3, produced at a syrup-to-pumpkin ratio of 1.27:1.00, showed intermediate hardness and maintained structural integrity during storage, supporting its selection as the preferred formulation, together with its favorable microstructural and organoleptic characteristics.

### 3.5. Changes in the Moisture Content of Candied Fruits During Storage

The moisture content of candied pumpkin samples stored in sealed polyamide packaging at 20 °C and 60% relative humidity decreased during the 9-month storage period (Table 6). At the beginning of storage, the Jerusalem artichoke syrup formulations generally contained more moisture than the sucrose-based control, with significant differences observed for Samples 2–5 (*p* < 0.05). A gradual decline in moisture content was observed in all formulations. The reductions became statistically significant by months 6 and 9 compared with the initial values (*p* < 0.05). From month 1 onward, all samples produced with Jerusalem artichoke syrup retained significantly more moisture than the control (*p* < 0.05). At the end of storage, Samples 4 and 5 showed the highest moisture retention, while Samples 1–3 formed a lower moisture group. Nevertheless, all Jerusalem artichoke syrup formulations maintained higher moisture levels than the sucrose-based control (*p* < 0.05), indicating that the syrup contributed to improved moisture retention during storage.

The reduction in moisture content during storage is consistent with previously reported behavior of candied products, in which water migration and osmotic redistribution continue even under controlled storage conditions [59,60,61]. As moisture decreases, soluble solids become increasingly concentrated within the product matrix [62]. The composition of the osmotic agent influences water-binding capacity and moisture equilibrium because different carbohydrate systems exhibit distinct sorption properties [63]. Blanching conditions and the resulting tissue porosity can also affect moisture migration during subsequent storage [64].

The higher moisture retention observed in formulations containing Jerusalem artichoke syrup may be associated with differences in carbohydrate composition and interactions between syrup components and the pumpkin matrix. These results indicate that Jerusalem artichoke syrup can reduce moisture loss during storage compared with sucrose syrup, thereby supporting the structural stability of candied pumpkin.

### 3.6. Changes in Water Activity of Candied Fruits During Storage

**Figure 3:**
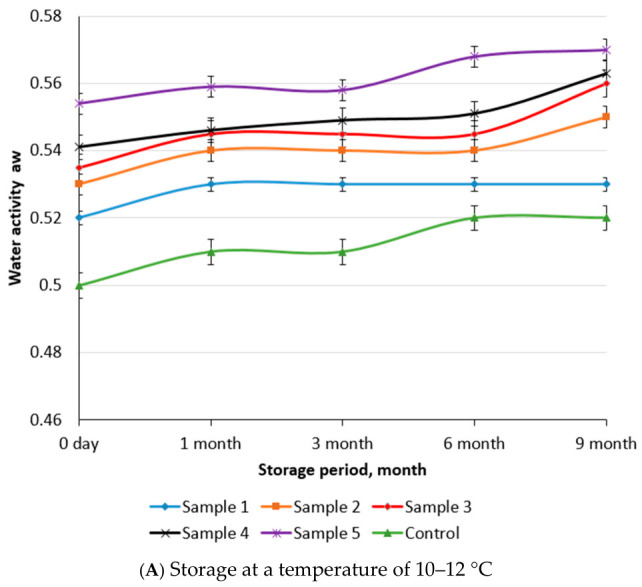

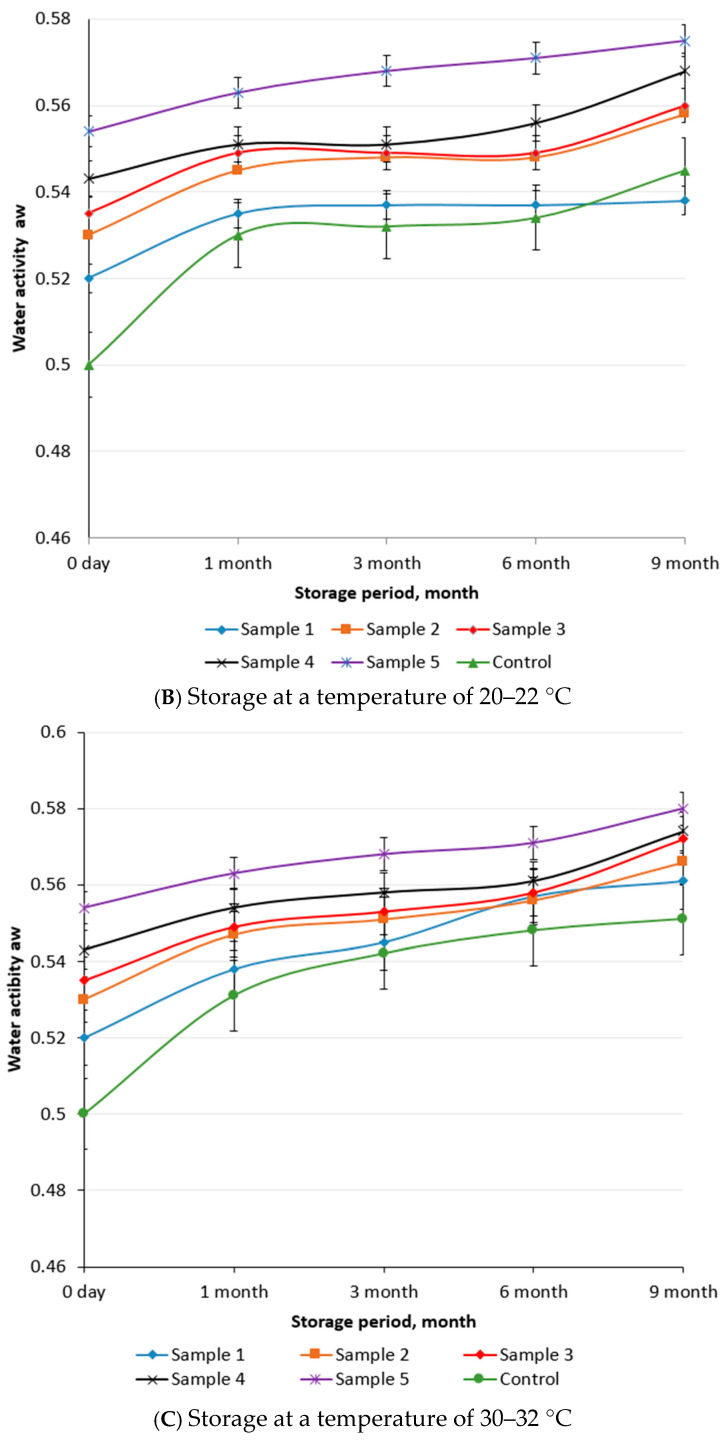
Diagram showing changes in water activity during storage of experimental and control samples of candied fruit at t = 10–12 °C, t = 20–22 °C, and t = 30–32 °C.

Water activity (a_w_) is a critical indicator of microbiological stability and depends on sugar composition, drying degree, and storage conditions [65,66]. During 9 months of storage in hermetically sealed packaging, all samples showed a gradual increase in a_w_ at each temperature regime (Figure 3). At 10–12 °C, the control increased from 0.50 to 0.52. Sample 3 increased from 0.535 to 0.56, while Sample 5 rose from 0.554 to 0.57. By month 9, Samples 4 and 5 demonstrated significantly higher a_w_ values compared with the control (*p* < 0.05). At 20–22 °C, the control increased from 0.50 to 0.545, whereas Sample 3 reached 0.56 and Sample 5 reached 0.575; differences between Sample 5 and the control were statistically significant (*p* < 0.05). At 30–32 °C, all samples showed a more pronounced increase; Sample 5 reached 0.58, significantly higher than the control (*p* < 0.05).

The gradual increase in a_w_ reflects internal moisture redistribution and equilibrium shifts within the sealed system. Even under hermetic conditions, temperature-driven diffusion processes promote moisture migration between the product core and surface [67]. Elevated temperature accelerates diffusion kinetics and may increase the permeability of packaging films [68]. Although a_w_ values approached 0.60 at higher temperatures, they remained below the critical threshold for most microbial growth, which supports microbiological safety [69].

Jerusalem artichoke syrup formulations exhibited slightly higher a_w_ compared with the sucrose-based control, likely due to differences in sugar composition and hygroscopic properties. Monosaccharide-rich systems may bind water differently than sucrose matrices, influencing sorption behavior [66]. Overall, storage at 10–12 °C and 20–22 °C maintained acceptable a_w_ levels, whereas 30–32 °C approached less favorable conditions for long-term stability.

### 3.7. Sensory Evaluation of Candied Pumpkin

In the next stage, organoleptic indicators were evaluated (Table 5). The organoleptic quality assessment was based on the perception of the characteristics of products by human senses, as well as on the use of established standards, and a color scale. The research results were expressed in points, which allows for a more objective assessment. Despite the fact that there is a subjective component in this type of quality analysis, depending on the qualification of the expert, the organoleptic method of research is an important stage for determining the suitability of food for consumption. Candied fruits are a confectionery product and can be stored for a long time. The change in organoleptic properties may be influenced by external factors, thereby changing their consumer properties [70]. Table 7 shows the descriptive analysis of the organoleptic properties of candied pumpkin.

**Figure 4:**
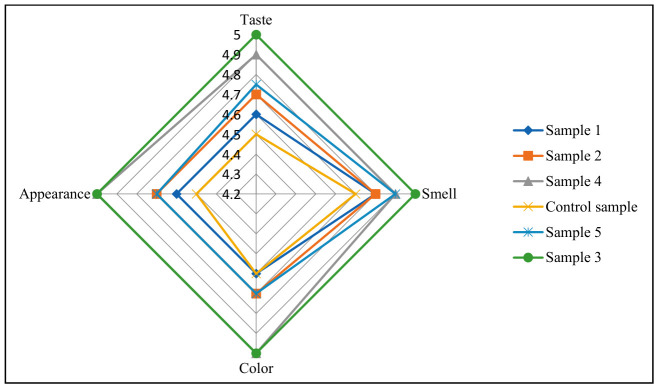
Organoleptic evaluation of candied pumpkin samples.

**Figure 5:**
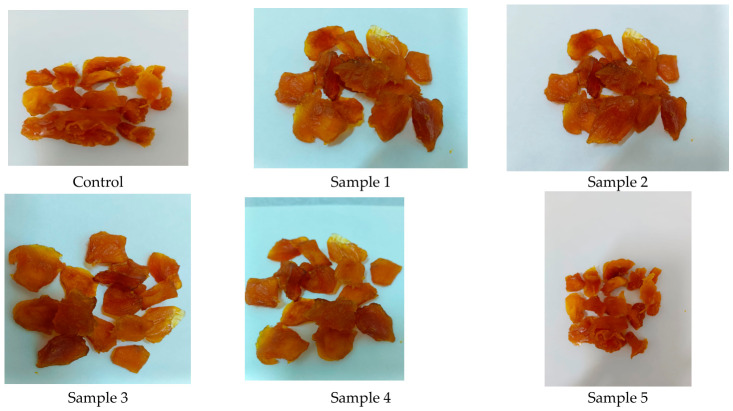
Finished candied fruits.

The organoleptic assessment revealed that samples produced with Jerusalem artichoke syrup generally scored higher than the sugar-based control, reflecting improvements in color, taste, and aroma (Figure 4). Among the experimental formulations, Samples 1 and 2 displayed notable deformation, whereas Sample 3 was distinguished by smooth, uniform pieces with a clean orange hue and a balanced pumpkin flavor subtly enhanced by the syrup. Experts found Sample 3 to most closely resemble the control in appearance and aroma, while offering a more favorable taste profile. The reduced caloric content of the Jerusalem artichoke syrup formulations contributed to positive consumer perception, providing an advantage in overall acceptability. In particular, Sample 3 demonstrated a combination of desirable organoleptic properties, along with lower caloric density, leading to its selection for further safety evaluations. The dried nature of candied pumpkins, whether produced with sugar syrup or Jerusalem artichoke syrup, facilitates extended shelf life by reducing water activity, although the syrup-to-pumpkin ratio appears to be a key factor influencing the final product’s sensory appeal (Figure 5).

Pumpkin candied with Jerusalem artichoke syrup is a confectionery product that can be called a concentrated product since it is dried. The amount of moisture decreases during the drying process, and the growth of microbial cells stops; as a result, they can be stored for a long time.

### 3.8. Vitamin Composition

The vitamin composition of Sample 3 and the sucrose-based control is presented in Table 8. Sample 3 contained significantly higher levels of retinol, pantothenic acid, vitamin C, and niacin than the control (*p* < 0.05), whereas β-carotene, thiamine, riboflavin, pyridoxine, folic acid, and α-tocopherol did not differ significantly (*p* ≥ 0.05). This indicates that Jerusalem artichoke syrup did not uniformly increase all vitamins but selectively influenced compounds that may have been contributed by the syrup or better retained during processing.

The increase in vitamin C is especially relevant because ascorbic acid is sensitive to oxygen, heat, and drying conditions and is often used as an indicator of nutritional quality in processed fruits and vegetables [71]. Osmotic dehydration can reduce water activity and help preserve quality, but it also involves simultaneous water loss, solute uptake, and migration of soluble compounds [72]. Therefore, the final vitamin profile likely reflects both the composition of the syrup and mass-transfer processes during impregnation and drying.

The unchanged β-carotene and α-tocopherol levels suggest that replacing sucrose syrup did not strongly affect fat-soluble compounds, which were mainly associated with the pumpkin matrix. Overall, Jerusalem artichoke syrup improved selected vitamin indicators, particularly vitamin C, pantothenic acid, and niacin, while maintaining the main carotenoid and tocopherol profile of candied pumpkin.

### 3.9. Mineral Composition

The mineral composition of Sample 3 and the sucrose-based control is presented in Table 9. Replacing sucrose syrup with Jerusalem artichoke syrup selectively affected the mineral profile rather than producing a general increase in all elements. Sample 3 contained significantly higher levels of magnesium, sodium, phosphorus, and iron than the control (*p* < 0.05), with phosphorus showing the most pronounced difference. In contrast, potassium and calcium showed only numerical increases, while sulfur, chlorine, manganese, copper, fluorine, and zinc remained comparable between the two formulations (*p* ≥ 0.05).

Unlike vitamins, minerals are not degraded by heat or drying; therefore, the observed differences are more likely to be related to raw-material composition and mass transfer during osmotic treatment. During soaking, the concentrated Jerusalem artichoke syrup acted as a hypertonic medium, promoting water removal from pumpkin tissue and diffusion of soluble syrup components into the product. The higher phosphorus and iron contents in Sample 3 may therefore reflect the mineral contribution of Jerusalem artichoke syrup and improved retention of water-soluble mineral compounds during processing. Jerusalem artichoke tubers contain mineral elements such as potassium, phosphorus, calcium, magnesium, and iron, which may contribute to the mineral profile of products derived from this raw material [73]. Overall, Jerusalem artichoke syrup improved selected mineral indicators, particularly phosphorus, magnesium, sodium, and iron, while maintaining a mineral profile broadly comparable to that of the sucrose-based control.

### 3.10. Microbiological Parameters

The microbiological stability of candied pumpkin was evaluated during storage in sealed packaging at 18 °C and a relative humidity of 60% (Table 10). Sample 3, produced with Jerusalem artichoke syrup, was compared with the sugar-based control over a 12-month period.

The total aerobic mesophilic count (TAMC) increased gradually during storage in both formulations (*p* < 0.05). In Sample 3, TAMC increased from 31 CFU/g after 7 days to 97 CFU/g after 12 months, while in the control sample it increased from 41 to 107 CFU/g. At 7 days, 1 month, 9 months, and 12 months, TAMC was significantly lower in Sample 3 than in the control (*p* < 0.05). At 6 months, the difference between the two formulations was not significant (*p* ≥ 0.05). All TAMC values remained substantially below the permissible limit of 500,000 CFU/g throughout storage.

Molds were not detected in either formulation during the first month of storage. After 6 months, mold counts reached 25 CFU/g in Sample 3 and 36 CFU/g in the control, with significantly lower values in Sample 3 (*p* < 0.05). A similar difference was observed at 9 and 12 months, when mold counts increased to 38 and 84 CFU/g in Sample 3 and to 69 and 112 CFU/g in the control, respectively (*p* < 0.05). Mold counts increased significantly during storage in both formulations from 6 to 12 months (*p* < 0.05). Sample 3 remained within the permissible limit of 100 CFU/g throughout storage, whereas the control exceeded this limit after 12 months.

Yeasts were also not detected during the first month. At 6 months, yeast counts reached 14 CFU/g in Sample 3 and 26 CFU/g in the control, with significantly lower values in Sample 3 (*p* < 0.05). At 9 months, the values increased to 28 and 47 CFU/g, respectively, and remained below the permissible limit of 50 CFU/g; the difference between formulations was also significant (*p* < 0.05). At 12 months, yeast counts increased to 64 CFU/g in Sample 3 and 73 CFU/g in the control, exceeding the established limit in both formulations. However, Sample 3 still showed a significantly lower yeast count than the control (*p* < 0.05). Yeast counts increased significantly during storage from 6 to 12 months in both formulations (*p* < 0.05).

Coliform bacteria and pathogenic microorganisms, including *Salmonella*, were not detected in either formulation at any sampling point. The lower mold and yeast counts observed in Sample 3 indicate that replacing sucrose syrup with Jerusalem artichoke syrup did not compromise microbiological stability and may contribute to improved storage performance. Similar storage-related increases in microbial counts have been reported for candied pumpkin, while osmotic treatment and protective packaging can restrict microbial growth [59]. Considering the microbiological limits together with the observed changes in moisture content, water activity, and hardness, the recommended shelf life of candied pumpkin is 9 months under the tested storage conditions.

## 4. Conclusions

Replacing sucrose with Jerusalem artichoke syrup in candied pumpkin production improved the nutritional and sensory characteristics of the product without compromising microbiological safety. All syrup-based formulations with syrup-to-pumpkin ratios of 1.25:1.00–1.30:1.00 contained more protein and fewer carbohydrates than the sucrose-based control. Sample 3, produced at a ratio of 1.27:1.00, showed the most balanced combination of physicochemical, structural, and organoleptic properties. This formulation also contained higher levels of vitamin C, pantothenic acid, niacin, phosphorus, magnesium, sodium, and iron than the control. During storage, water activity remained below 0.60, and microbiological indicators remained within permissible limits for 9 months. Further research may include determination of the glycemic index or predicted glycemic response of the final candied product, as well as evaluation of antioxidant activity and bioactive-compound retention during storage. Additional studies using different packaging systems, larger consumer panels, and pilot-scale production would help confirm the technological feasibility and commercial potential of candied pumpkin produced with Jerusalem artichoke syrup.

## 5. Patents

The developed candied fruits were used as an ingredient under the patent for utility model RK No. 6722 Curd product. Bulletin number 48, Bulletin date: 3 December 2021. Authors: Galimova A.M., Kosheleva E.A., Smolnikova F.H.

## Figures and Tables

**Table 1:** Formulation of candied pumpkin samples produced with Jerusalem artichoke syrup and sucrose syrup.

Name of Raw Materials	Quantity, g					
	Sample 1 (JAS 1.30:1)	Sample 2 (JAS 1.29:1)	Sample 3 (JAS 1.27:1)	Sample 4 (JAS 1.26:1)	Sample 5 (JAS 1.25:1)	Control (Sucrose Syrup)
Syrup-to-pumpkin ratio (*w*/*w*)	1.30:1.00	1.29:1.00	1.27:1.00	1.26:1.00	1.25:1.00	-
Pumpkin, peeled (g)	1005	1010	1020	1025	1030	1000
Jerusalem artichoke syrup (g)	1315	1310	1300	1295	1290	
Sugar (g)	-	-	-	-	-	720
Water (g)	-	-	-	-	-	600

Values for pumpkin, syrup, sugar, and water are expressed in grams (g). The syrup-to-pumpkin ratio is expressed as a mass ratio (*w*/*w*). “-” indicates that the ingredient was not used in the corresponding formulation.

**Table 2:** Chemical and physicochemical characteristics of raw materials.

Parameter	Fresh Pumpkin	Jerusalem Artichoke Syrup	Sucrose Syrup
Moisture, g/100 g	87.1 ± 2.15 ^c^	26.9 ± 0.70 ^a^	32.1 ± 1.03 ^b^
Dry matter, g/100 g	12.3 ± 0.35 ^a^	73.0 ± 1.90 ^c^	67.2 ± 1.81 ^b^
Protein, g/100 g	1.33 ± 0.02	nd	nd
Fat, g/100 g	0.30 ± 0.00 ^b^	0.01 ± 0.00 ^a^	nd
Ash, g/100 g	0.78 ± 0.01 ^b^	2.00 ± 0.05 ^c^	0.05 ± 0.00 ^a^
Total carbohydrates, g/100 g	8.63 ± 0.12 ^a^	65.8 ± 1.41 ^b^	68.6 ± 1.18 ^b^
Dietary fiber, g/100 g	1.20 ± 0.02	nd	nd
Inulin/fructans, g/100 g	nd	5.00 ± 0.10	nd
Energy value, kcal/100 g	30 ± 1 ^a^	250 ± 7 ^b^	270 ± 9 ^c^
pH	6.2 ± 0.2 ^b^	5.1 ± 0.1 ^a^	6.7 ± 0.2 ^c^
Water activity, a_w_	0.99 ± 0.03 ^b^	0.85 ± 0.02 ^a^	0.83 ± 0.02 ^a^
Density, g/cm^3^	1.03 ± 0.03 ^a^	1.36 ± 0.04 ^b^	1.33 ± 0.04 ^b^
Viscosity, mPa·s	452 ± 12 ^a^	3982 ± 109 ^c^	1803 ± 54 ^b^

Different superscript letters within the same row indicate significant differences among formulations (*p* < 0.05). nd—not detected.

**Table 3:** Bioactive compounds and mineral composition of fresh pumpkin and Jerusalem artichoke syrup.

Parameter	Fresh Pumpkin	Jerusalem Artichoke Syrup
Vitamin C, mg/100 g	9.5 ± 0.2 ^a^	93.8 ± 2.4 ^b^
β-Carotene, mg/100 g	5.2 ± 0.15 ^b^	0.012 ± 0.001 ^a^
Total phenolic compounds, mg GAE/100 g	120 ± 4 ^b^	80 ± 2 ^a^
Calcium, mg/100 g	25.8 ± 0.7 ^b^	17.4 ± 0.5 ^a^
Phosphorus, mg/100 g	31.9 ± 1.0 ^a^	79.9 ± 2.3 ^b^
Magnesium, mg/100 g	16.2 ± 0.4 ^b^	12.4 ± 0.4 ^a^
Potassium, mg/100 g	298.5 ± 7.90 ^b^	205.6 ± 5.41 ^a^
Iron, mg/100 g	0.61 ± 0.01 ^b^	0.41 ± 0.01 ^a^

Different superscript letters within the same row indicate significant differences among formulations (*p* < 0.05).

**Table 4:** Chemical composition of the candied pumpkins with Jerusalem artichoke syrup, including candied pumpkins with sugar syrup as a control sample.

Sample	Proteins, g/100 g	Fats, g/100 g	Carbohydrates, g/100 g	Moisture, g/100 g	Ash, g/100 g	Caloric Content, kcal/100 g
Control sample	0.40 ± 0.01 ^a^	Not detected	40.40 ± 0.50 ^c^	20.0 ± 0.38 ^a^	0.65 ± 0.01 ^a^	163.20 ± 1.71 ^c^
Sample 1	0.99 ± 0.01 ^b^	0.22 ± 0.00 ^c^	23.35 ± 0.31 ^b^	23.3 ± 0.54 ^b^	0.66 ± 0.01 ^a^	99.34 ± 1.68 ^b^
Sample 2	0.98 ± 0.02 ^b^	0.21 ± 0.00 ^bc^	23.17 ± 0.29 ^b^	22.4 ± 0.39 ^b^	0.67 ± 0.01 ^a^	98.49 ± 0.84 ^b^
Sample 3	0.97 ± 0.01 ^b^	0.20 ± 0.00 ^b^	22.15 ± 0.29 ^ab^	20.8 ± 0.25 ^a^	0.68 ± 0.01 ^a^	94.28 ± 1.25 ^ab^
Sample 4	0.97 ± 0.02 ^b^	0.18 ± 0.00 ^a^	21.79 ± 0.41 ^a^	21.1 ± 0.37 ^a^	0.68 ± 0.01 ^a^	92.66 ± 0.80 ^a^
Sample 5	0.96 ± 0.01 ^b^	0.17 ± 0.00 ^a^	21.25 ± 0.31 ^a^	20.1 ± 0.26 ^a^	0.68 ± 0.01 ^a^	90.37 ± 1.09 ^a^
*p*-value	<0.001	<0.001	<0.001	<0.01	>0.25	<0.001

^a–c^ Different superscript letters within the same column indicate significant differences among formulations (*p* < 0.01).

**Table 5:** Changes in the hardness of candied fruits during storage.

Sample	0 Day	1 Month	3 Month	6 Month	9 Month
Sample 1	40 ± 1 ^Ca^	41 ± 1 ^Ca^	42 ± 1 ^Cab^	44 ± 1 ^Cbc^	47 ± 1 ^Cc^
Sample 2	38 ± 1 ^Ca^	39 ± 1 ^Ca^	40 ± 1 ^Cab^	43 ± 1 ^Cb^	45 ± 1 ^BCb^
Sample 3	35 ± 1 ^Ba^	36 ± 1 ^Ba^	37 ± 1 ^Bab^	39 ± 1 ^Bb^	44 ± 1 ^Bc^
Sample 4	34 ± 1 ^Ba^	35 ± 1 ^Ba^	36 ± 1 ^Bab^	38 ± 1 ^ABb^	42 ± 1 ^ABc^
Sample 5	30 ± 0.5 ^Aa^	30 ± 0.6 ^Aa^	32 ± 1 ^Aa^	36 ± 1 ^Ab^	40 ± 1 ^Ac^
Control	45 ± 1 ^Da^	45 ± 1 ^Da^	47 ± 1 ^Dab^	49 ± 1 ^Db^	55 ± 1 ^Dc^

Different uppercase superscript letters within the same column indicate significant differences among formulations at the same storage time (*p* < 0.05). Different lowercase superscript letters within the same row indicate significant differences among storage periods for the same formulation (*p* < 0.05).

**Table 6:** Moisture content of candied pumpkin samples during storage.

Sample	0 Day	1 Month	3 Month	6 Month	9 Month
Sample 1	21.25 ± 0.40 ^ABb^	21.01 ± 0.33 ^Bb^	20.85 ± 0.37 ^Bb^	19.5 ± 0.35 ^Ba^	18.75 ± 0.16 ^Ba^
Sample 2	21.79 ± 0.40 ^Bb^	21.05 ± 0.24 ^Bb^	20.54 ± 0.32 ^Bab^	19.93 ± 0.31 ^BCa^	19.49 ± 0.26 ^Ba^
Sample 3	22.15 ± 0.49 ^BCc^	21.85 ± 0.44 ^BCbc^	21.65 ± 0.50 ^BCbc^	20.40 ± 0.36 ^BCab^	19.75 ± 0.32 ^Ba^
Sample 4	23.17 ± 0.55 ^Cc^	22.74 ± 0.48 ^CDbc^	22.52 ± 0.35 ^Cbc^	21.23 ± 0.41 ^CDab^	20.87 ± 0.34 ^Ca^
Sample 5	23.35 ± 0.51 ^Cc^	23.01 ± 0.47 ^Dbc^	22.16 ± 0.27 ^Cb^	21.78 ± 0.38 ^Dab^	20.85 ± 0.41 ^Ca^
Control	20.01 ± 0.26 ^Ac^	19.3 ± 0.34 ^Ac^	18.78 ± 0.31 ^Abc^	18.02 ± 0.32 ^Aa^	17.5 ± 0.19 ^Aa^

Different uppercase superscript letters within the same column indicate significant differences among formulations at the same storage time (*p* < 0.05). Different lowercase superscript letters within the same row indicate significant differences among storage periods for the same formulation (*p* < 0.05).

**Table 7:** Organoleptic indicators of candied pumpkins.

Indicator	Sample 1	Sample 2	Sample 3	Sample 4	Sample 5	Control Sample
Appearance	Candied fruit pieces of uniform shape with pronounced deformation	Candied fruit pieces with a uniform shape with a slightly pronounced deformation	Homogeneous smooth pieces of candied fruits	Candied fruit pieces with a uniform shape	Candied fruit pieces with a uniform shape	Homogeneous, shape-preserving
Taste	Smooth pumpkin flavor with a pronounced aftertaste of Jerusalem artichoke syrup	Homogeneous pumpkin flavor with a moderately pronounced flavor of Jerusalem artichoke syrup	Pure, homogeneous pumpkin flavor with a slight aftertaste of Jerusalem artichoke syrup in all pieces, without foreign flavors	Pumpkin flavor with uneven Jerusalem artichoke flavor	Pumpkin heterogeneous taste with a slightly pronounced aftertaste of Jerusalem artichoke syrup	The taste is characteristic of pumpkin, sweet
Color	Light brown	Cream, light brown	Orange	Faintly orange	Yellowish-orange	Orange, brownish
Smell	Pure, strongly sweet, with a predominance of the smell of Jerusalem artichoke	Pure moderately sweet, slightly pumpkin	Clean, sweet, evenly pumpkin in all pieces	Pumpkin, heterogeneous, slightly sweet	Pumpkin, slightly pronounced sweet	Pleasant, sweet, pumpkin

Control sample—candied pumpkins with sugar syrup.

**Table 8:** Vitamin composition of pumpkin candied fruits, mg/100 g.

Vitamins	Control	Sample 3
A, retinol, mg	0.374 ± 0.007 ^a^	0.391 ± 0.009 ^b^
β-carotene, mg	2.247 ± 0.03 ^a^	2.307 ± 0.04 ^a^
B_1_, thiamine, mg	0.067 ± 0.001 ^a^	0.068 ± 0.001 ^a^
B_2_, riboflavin, mg	0.085 ± 0.002 ^a^	0.086 ± 0.001 ^a^
B_5_, pantothenic acid, mg	0.631 ± 0.01 ^a^	1.004 ± 0.01 ^b^
B_6_, pyridoxine, mg	0.205 ± 0.004 ^a^	0.205 ± 0.003 ^a^
B_9_, folic acid, mg	0.022 ± 0.001 ^a^	0.022 ± 0.002 ^a^
C, ascorbic acid, mg	5.05 ± 0.07 ^a^	8.04 ± 0.13 ^b^
E,α-tocopherol, TE, mg	0.631 ± 0.001 ^a^	0.654 ± 0.001 ^a^
B_3,_ niacin, mg	0.67 ± 0.01 ^a^	1.03 ± 0.01 ^b^

The mean ± SD (standard deviation) within rows with different small letters differs significantly (*p* < 0.05). Control sample—candied pumpkins with sugar syrup.

**Table 9:** Mineral composition of pumpkin candied fruits, mg/100 g.

Macromineral	Control	Sample
Potassium, K, mg	319.7 ± 5.9 a	338.7 ± 5.5 a
Calcium, Ca, mg	39.34 ± 0.52 a	41.03 ± 0.84 a
Magnesium, Mg, mg	20.97 ± 0.25 a	22.13 ± 0.22 b
Sodium, Na, mg	6.62 ± 0.14 a	7.44 ± 0.11 b
Sulfur, S, mg	28.39 ± 0.57 a	28.40 ± 0.59 a
Phosphorus, P, mg	36.7 ± 0.52 a	56.7 ± 0.79 b
Chlorine, Cl, mg	29.96 ± 0.53 a	29.96 ± 0.42 a
Microelement		
Iron, Fe, mg	0.73 ± 0.01 a	0.82 ± 0.01 b
Manganese, Mn, mcg	0.06 ± 0.00 a	0.06 ± 0.00 a
Copper, Cu, mcg	283.8 ± 3.8 a	284.1 ± 2.5 a
Fluorine, F, mcg	135.6 ± 1.6 a	135.7 ± 1.7 a
Zinc, Zn, mg	0.37 ± 0.01 a	0.38 ± 0.01 a

The mean ± SD (standard deviation) within rows with different small letters differs significantly (*p* < 0.05). Control sample—candied pumpkins with sugar syrup.

**Table 10:** Microbiological quality of candied pumpkin during storage at 18 °C and 60% relative humidity.

Microbiological Indicators	Type of Sample	The Value of Microbiological Indicators					
		Limit, TR CU 021/2011 [74]	7 Days	1 Month	6 Months	9 Months	12 Months
TAMC, CFU/g	Sample 3	≤500,000	31 ± 1 ^Aa^	52 ± 1 ^Ab^	71 ± 1 ^Ac^	83 ± 2 ^Ad^	97 ± 2 ^Ae^
	Control sample		41 ± 1 ^Ba^	64 ± 1 ^Bb^	73 ± 1 ^Ac^	91 ± 2 ^Bd^	107 ± 3 ^Be^
Mold, CFU/g	Sample 3	≤100	ND	ND	25.0 ± 0.3 ^Aa^	38.0 ± 0.5 ^Ab^	84.0 ± 1.8 ^Ac^
	Control sample		ND	ND	36.0 ± 0.5 ^Ba^	69.0 ± 1.0 ^Bb^	112.0 ± 2.4 ^Bc^
Yeast, CFU/g	Sample 3	≤50	ND	ND	14.0 ± 0.2 ^Aa^	28.0 ± 0.8 ^Ab^	64.0 ± 1.4 ^Ac^
	Control sample		ND	ND	26.0 ± 0.4 ^Ba^	47.0 ± 1.1 ^Bb^	73.0 ± 1.6 ^Bc^
Coliform bacteria, not allowed in 1.0 g	Sample 3	Absence	ND	ND	ND	ND	ND
	Control sample		ND	ND	ND	ND	ND
*Salmonella* spp., not allowed in 25 g	Sample 3	Absence	ND	ND	ND	ND	ND
	Control sample		ND	ND	ND	ND	ND

Values are presented as mean ± SD (*n* = 3). ND, not detected. TAMC, total aerobic mesophilic count. Different uppercase superscript letters within the same column indicate significant differences between samples at the same storage time (*p* < 0.05). Different lowercase superscript letters within the same row indicate significant differences during storage for the same sample (*p* < 0.05). Values sharing the same superscript letter do not differ significantly.

## Data Availability

The original contributions presented in this study are included in the article. Further inquiries can be directed to the corresponding author.
